# Supplementary material for: βIII-Tubulin Structural Domains Regulate Mitochondrial Network Architecture in an Isotype-Specific Manner
Source: Cells. 2022 Feb 23;11(5):776. doi: 10.3390/cells11050776 (PMC8909761; doi:10.3390/cells11050776)
Supplement: Supplementary file 1 [file cells-11-00776-s001.zip › cells-1584043-supplementary.pdf]

cells MDPI

Supplementary Figures

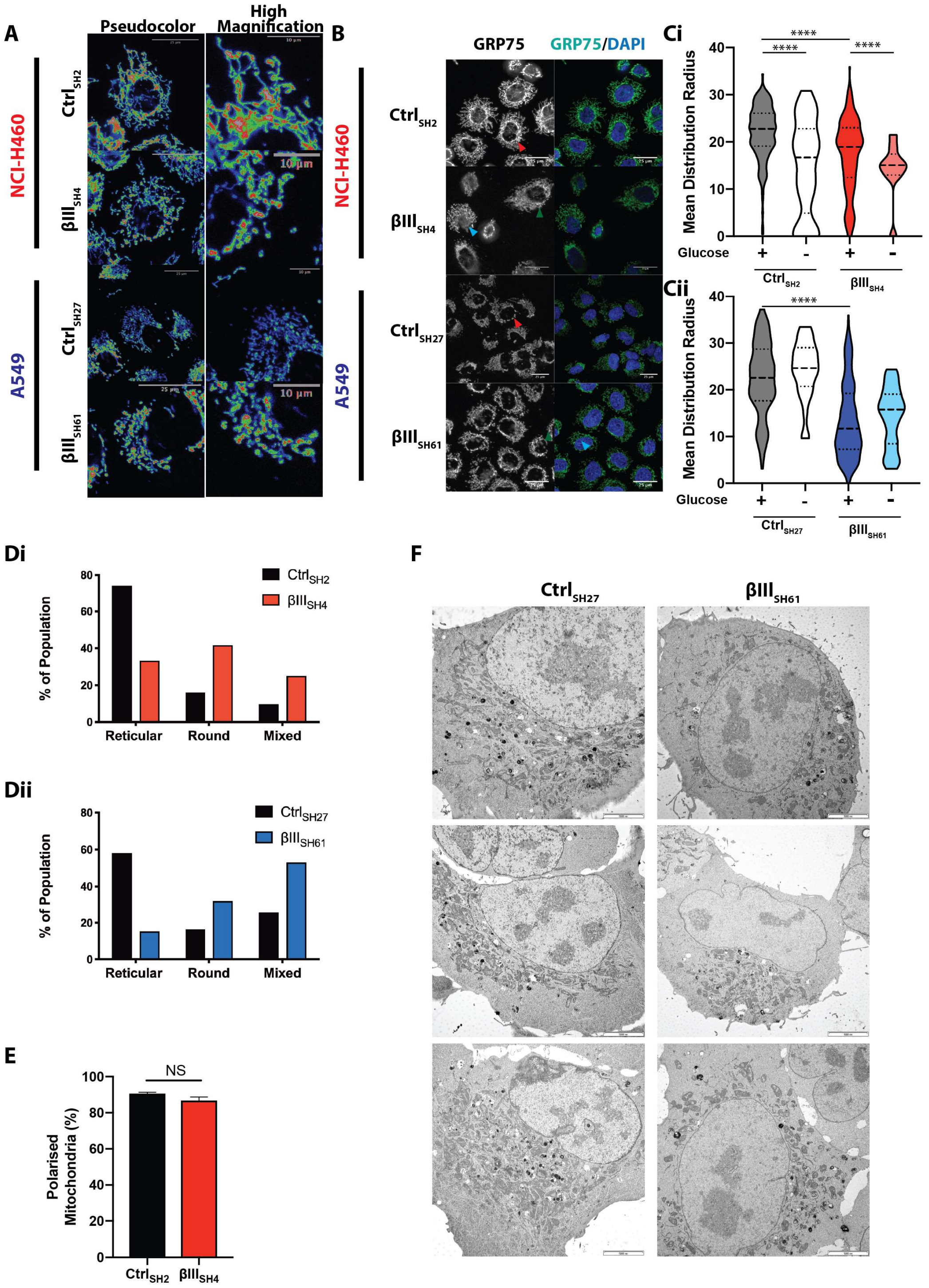

Supplementary Figure S1. βIII-Tubulin regulates mitochondrial network structure and morphology in glucose-starved cells. (**A**) Pseudocolored GRP75 staining intensity at single cell (left, scale bar 25 µm) and high magnification (right, scale bar 10 µm) corresponding to Figure 1A demonstrating the mitochondrial network architecture in NCI-H460 and A549 cells expressing control (H460 $Ctrl_{SH2}$, A549 $Ctrl_{SH27}$) and βIII-tubulin targeted shRNA (H460 $βIII_{SH4}$, A549 $βIII_{SH61}$). (**B**)

Mitochondrial network distribution by GRP75 immunostaining (left panel: greyscale; right panel: GRP75 green, DAPI blue) in NCI-H460 and A549 cells expressing control (H460 $Ctrl_{SH2}$, A549 $Ctrl_{SH27}$) and βIII-tubulin targeted shRNA (H460 $βIII_{SH4}$, A549 $βIII_{SH61}$). Red arrowhead: reticular mi-tochondrial morphology; blue arrowhead: rounded morphology; green arrowhead: mixed mor-phology. Scale bars 25μm. Representative of n=3 independent experiments. (**C**) Mitochondrial mean distribution radius in NCI-H460 (i) and A549 (ii) cells. Data from the analysis of 30-60 cells from n=3 independent experiments. ****p<0.0001 Kruskal-Wallis test with Dunn's multiple comparison cor-rection. (**D**) The proportion of cells with rounded, reticular or mixed mitochondrial morphology in NCI-H460 (**i**) and A549 (**ii**) cells. Data from at least 50 cells from each of 2 independent experiments. (**E**) Mitochondrial membrane potential in NCI-H460 cells expressing control ($Ctrl_{SH2}$) and βIII-tubu-lin targeted shRNA (H460 $βIII_{SH4}$). NS: not significant. (**F**) Representative uncoloured electron micro-graphs of A549 cells expressing control (A549 $Ctrl_{SH27}$) and βIII-tubulin targeted shRNA (A549 $βIII_{SH61}$) corresponding to pseudocoloured micrographs in Figure 2A.

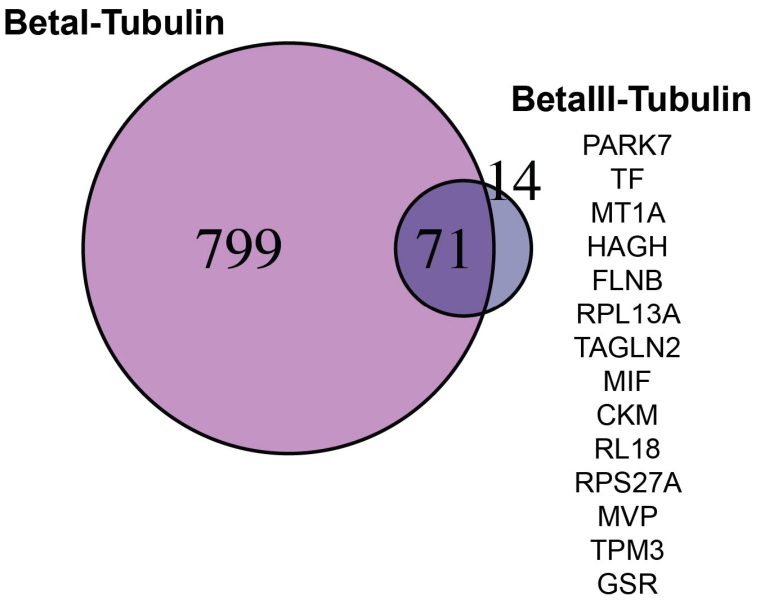

Supplementary Figure S2. βIII-Tubulin interacts with mitochondrial regulators. (**A**) Interacting pro-teins identified in qualitative mass spectrometry proteomics of βI-tubulin and βIII-tubulin immuno-precipitation.

Supplementary Table S1. Proteins identified as interacting with βIII-tubulin but not βI-tubulin.

Protein	Protein Name	Localisation	Function	Reference
PARK7	Parkinson disease protein 7	Under normal conditions located in the cytoplasm, and to a lesser extent in the nucleus and mitochondrion; translocates to the mitochondrion then nucleus in response to oxidative stress	Oxidative stress sensor, redox-sensitive chaperone, required for correct mitochondrial morphology and function	(Ozawa et al., 2020; Xiong et al., 2009)
TF	Tissue Factor	Activation of the clotting cascade;	Transmembrane protein at plasma membrane;	(Peña et al., 2012)

		Enhancing integrin signalling; Cytoskeletal reorganisation	Pena et al 2012 have shown that it is localised to the golgi and transported to the lamellopodia	
MT1A	Metallothionein 1A	Zinc homeostasis	Cytosolic; nucleus	(Borchert et al., 2020)
HAGH	Hydroxyacylglutathione hydrolase, mitochondrial	Thiolesterase that hydrolyses S-D-lactoyl glutathione to form glutathione	Mitochondria (matrix)	(Cordell et al., 2004)
FLNB	FFilamin-B	Connects cell membrane to the actin cytoskeleton	Cytoplasm/cell cortex	(Iguchi et al., 2015)
RPL13A	60S ribosomal protein L13A	Ribosomal and extra-ribosomal functions; transcriptional regulation	cytoplasm	(Mazumder et al., 2003)
TAGLN2	Transgelin-2	Regulation of actin cytoskeleton	Cytoplasm and extracellular	(Kim et al., 2021)
MIF	Macrophage Migration Inhibitory Factor	Proinflammatory cytokine	Cytoplasm and extracellular space	(Kleemann et al., 2000)
CKM	Creatine-Kinase M-type	Transfers phosphate between ATP and creatine	cytoplasm	(Kazak and Cohen, 2020)
RL18	60S ribosomal protein L18	translation	cytoplasm	(Behrmann et al., 2015)
RPS27A	Ubiquitin-40S ribosomal protein S27A	Cytoplasmic translation regulation	cytoplasm	(Montellese et al., 2020)
MVP	Major Vault Protein	Protein scaffold and nucleo-cytoplasmic transport	Cytoplasm and nucleus including perinuclear region	(Kim et al., 2006)
TPM3	Tropomyosin alpha-3 chain	Stabilizing actin filaments	cytoplasm	(Meiring et al., 2019)
GSR	Glutathione Reductase, mitochondrial	Redox homeostasis	Mitochondria and cytoplasm	(Kelner and Montoya, 2000)

Supplementary Methods

Co-Immunoprecipitation

Co-immunoprecipitation of βIII-tubulin and βI-tubulin were performed using the Chromotek GFP immunoprecipitation kit and Pierce™ Crosslink IP Kit (Thermo Fisher #26147), as described previously (Parker et al., 2016), according to the manufacturer's instructions.

For mass spectrometry analysis, gene-edited NCI-H460 cells were cultured for 48h before cell lysis and lysate clearing using the Chromotek GFP immunoprecipitation kit according to the manufacturer's instructions.

For validation of mass spectrometry results in NCI-H460 Parental lines by western blotting, 10 μg of purified, mouse monoclonal anti-βIII-tubulin Antibody (TUJ1, Biolegend #801202) was coupled to protein A/G Plus agarose resin and cross-linked using the supplied DSS reagent. NCI-H460 Parental cells were grown to near confluency in a T75 flask, cells were then washed with warm PBS before undergoing cell lysis. Lysis was performed on ice using the supplied IP lysis buffer for 10 mins with regular, gentle mixing. Following centrifugation, lysates were cleared and loaded onto the column containing the cross-linked, mouse anti-βIII-tubulin Antibody for 2 hours at 4°C for immunoprecipitation to occur. Following several washes with TBS, bound antigens were eluted from the column, and eluates were analysed by western blotting to confirm presence of the antigen using a Rabbit polyclonal anti-βIII-tubulin Antibody (Biolegend #802001), and to detect the Co-immunoprecipitated PARK7 (clone DJ-1) using a Rabbit monoclonal anti-PARK7/DJ-1 antibody (Cell Signaling Technologies #5933).

Mass Spectrometry

Immunoprecipitated protein samples were prepared as described previously (Shevchenko et al., 1996). Briefly, protein samples were reduced (5mM DTT at 37°C for 30 min), alkylated (10mM iodoacetamide for RT for 30 min in the dark) followed by tryptic digestion overnight at 37C. Digest peptides were separated by nano-LC using an Ultimate 3000 HPLC and autosampler system (Dionex, Amsterdam, Netherlands). Samples (2.5 μl) were concentrated and desalted onto a micro C18 precolumn (300 μm x 5 mm, Dionex) with H_2O:CH_3CN (98:2, 0.05 % TFA) at 15 μl/min. After a 4 min wash the pre-column was switched (Valco 10 port valve, Dionex) into line with a fritless nano column (75μ x ~10cm) containing C18 media (1.9 μ, 120Å, Dr Maisch, Ammerbuch-Entringen Germany). Peptides were eluted using a linear gradient of H_2O:CH_3CN (98:2, 0.1 % formic acid) to H_2O:CH_3CN (64:36, 0.1 % formic acid) at 200 nl/min over 30 min. High voltage (2000 V) was applied to low volume tee (Upchurch Scientific) and the column tip positioned approximately 0.5 cm from the heated capillary (T=275°C) of an Orbitrap Velos (Thermo Electron, Bremen, Germany) mass spectrometer. Positive ions were generated by electrospray and the Orbitrap operated in data dependent acquisition mode (DDA).

A survey scan m/z 350-1750 was acquired in the Orbitrap (Resolution = 30,000 at m/z 400, with an accumulation target value of 1,000,000 ions) with lockmass enabled. Up to the 10 most abundant ions (>4,000 counts) with charge states > +2 were sequentially isolated and fragmented within the linear ion trap using collisionally induced dissociation with an activation q = 0.25 and activation time of 30 ms at a target value of 30,000 ions. M/z ratios selected for MS/ MS were dynamically excluded for 30 seconds.

All MS/MS spectra were searched against the Uniprot database using MASCOT (version 2.3) with the following search criteria: enzyme specificity was trypsin; precursor and product ion tolerances were at 4 ppm and ± 0.4 Da, respectively; variable modification of methionine oxidation; and one missed cleavage was allowed. Proteins identified with at least two unique peptides were retained for further analysis. Mass spectrometric analysis

was carried out at the Bioanalytical Mass Spectrometry Facility, University of New South Wales, Australia.

Proteins identified as associated with βI-tubulin or βIII-tubulin were submitted to Stringdb (https://string-db.org/) to identify protein interaction networks specific to the βIII-tubulin isotype. These same proteins that specifically associate with βIII-tubulin were submitted to DAVID Functional Annotation Analysis (Huang et al., 2009) to identify pathways enriched for these βIII-tubulin-associating pathways. P-values were adjusted for multiple comparisons using the Bonferroni method.
